# Predictive factors of symptomatic lumbar pseudoarthrosis following multilevel primary lumbar fusion

**DOI:** 10.1016/j.xnsj.2023.100302

**Published:** 2023-12-05

**Authors:** Hania Shahzad, Moizzah Ahmad, Varun K. Singh, Nazihah Bhatti, Elizabeth Yu, Frank M. Phillips, Safdar N. Khan

**Affiliations:** a The Ohio State University Wexner Medical Center, 410 W 10th Ave, Columbus, OH, 43210, United States; b Wexner Medical Center, 410 W 10th Avenue, Columbus OH, 43210, United States; c Rush University Medical Center, Department of Orthopedics, 1620 W Harrison St, Chicago, IL 60612, United States

**Keywords:** Lumbar pseudoarthrosis, Spinal fusion, Predictive factors, Comorbidities, Surgical approaches, Substance use disorders

## Abstract

**Background:**

Lumbar spinal fusion surgery is a well-established treatment for various spinal disorders. However, one of its complications, pseudoarthrosis, poses a significant concern. This study aims to explore the incidence, time and predictive factors contributing to pseudoarthrosis in patients who have undergone lumbar fusion surgery over a 10-year period.

**Methods:**

Data for this research was sourced from the PearlDiver database where insurance claims of patients who underwent multilevel lumbar spinal fusion between 01/01/2010 and 10/31/2022 were examined for claims of pseudoarthrosis within the 10 years of their index procedure. A variety of demographic, comorbid, and surgical factors were assessed, including age, gender, Elixhauser Comorbidity Index (ECI), surgical approach, substance use disorders and history of spinal disorders. Statistical analyses, including chi-squared tests, multivariate analysis, and cox survival analysis were employed to determine significant associations.

**Results:**

Among the 76,337 patients included in this retrospective study, 2.70% were diagnosed with symptomatic lumbar pseudoarthrosis at an average of 7.38 years in a 10-year follow-up. Multivariate and Cox hazard analyses revealed that significant predictors of symptomatic pseudoarthrosis development following multilevel primary lumbar fusion include vitamin D deficiency, osteoarthritis, opioid and NSAID use, tobacco use, and a prior history of congenital spine disorders.

**Conclusions:**

In summary, this study revealed a 2.70% incidence of symptomatic lumbar pseudoarthrosis within 10 years of the index procedure. It highlighted several potential predictive factors, including comorbidities, surgical approaches, and substance use disorders, associated with the development of symptomatic pseudoarthrosis. Future research should focus on refining our understanding of these factors to improve patient outcomes and optimize healthcare resource allocation.

## Background

Lumbar spinal fusion surgery is a well-established and effective treatment for a variety of spinal disorders [1]. However, this procedure is not without its challenges, and 1 significant complication is the development of pseudoarthrosis, which involves the failure to achieve a solid bony fusion, often within 1 year of the index procedure [2]. The reported incidence of pseudoarthrosis varies widely, ranging from 5% to 35% [3], with a higher prevalence observed in surgeries involving 3 or more spinal vertebrae [4,5]. Several factors have been identified as influencing the likelihood of developing pseudoarthrosis. Patients with higher body mass indices (BMIs), diabetes, and obesity [6] are at an increased risk of developing pseudoarthrosis. Lifestyle choices, such as smoking, have also been shown to significantly elevate the rates of pseudoarthrosis, with regular smokers facing rates as high as 40%, compared to 8% in nonsmokers [7]. Predicting when or if pseudoarthrosis will become symptomatic for the patient is often a complex task [2,8]. Nevertheless, when it does require management, it can lead to reoperations even up to 10 years after the initial procedure [9], [10], [11].

Symptomatic pseudoarthrosis may necessitate surgical intervention [12] and is a common reason for revision surgeries following lumbar fusion procedures [2,12]. However, the outcomes of such revision surgeries have shown inconsistent clinical results. Consequently, a critical focus has been placed on the prevention of pseudoarthrosis after the initial operation, with advancements in bone grafting materials, instrumentation, and surgical techniques proving to be the most effective strategies [13]. As the volume of fusion procedures continues to grow [14], understanding and mitigating the risk factors associated with symptomatic pseudoarthrosis is essential to improve clinical outcome of lumbar fusion surgeries.

The rationale for conducting this study lies in the absence of limited data available regarding risk factors for the development of symptomatic pseudoarthrosis, in patients who have undergone multilevel primary lumbar fusion surgery within a 10-year follow-up. Exploring these predictive factors holds substantial benefits for various stakeholders in the healthcare process. Patients considering surgery can make more informed decisions, surgeons can enhance preoperative planning by implementing protective measures, risk stratification can be optimized to tailor treatment, and the likelihood of revision surgery can be predicted, all contributing to improved patient outcomes.

## Methods

### Data source

The data was extracted from the PearlDiver database of over 41 billion HIPAA-compliant patient records which has claims based on the International Classification of Diseases (ICD)-9 and ICD-10 classifications. Claims for procedures are classified under the Current Procedural Terminology codes whereas claims for prescription and brand-name drugs are classified according to the Uniform System of Classification and the U.S. Food and Drug Administration National Drug Code Directory. This database was selected for analysis because it contains a large population of patients which reduces the risk of Type-II errors. Since the data is de-identified, ethical review board approval was not required for this study.

### Eligibility criteria

Patients who underwent multilevel lumbar spinal fusion between 01/01/2010 and 10/31/2022 were identified using the procedural codes for lumbar spinal fusion (see Appendix 1). All selected patients were initially filtered to ensure they had no prior lumbar fusion surgeries. Patients with any associated opioid use claims within 3 months prior to the day of index procedure were excluded to ensure that all included patients were opioid-naive. The resulting population was followed for a 10-year interval and were grouped into those that had a claim for pseudoarthrosis, that is, ICD-10-D-M960, and those who did not within the 10-year interval following the primary lumbar spinal fusion procedure.

### Study outcomes

The primary outcome was to evaluate the overall incidence and time of the development of pseudoarthrosis within 10 years following lumbar spinal fusion. Secondary outcomes included the demographic, comorbid, and surgical factors predictive of development of pseudoarthrosis in patients undergoing lumbar spinal fusion.

### Evaluated co-morbidities

The comorbid conditions that may be associated with increased odds of developing pseudoarthrosis were identified from the literature. Major demographic variables assessed included age, gender, Elixhauser Comorbidity Index (ECI) [15] and a diagnosis of obesity. Evaluated surgical variables included surgical levels, approach, and wound infections developed within 30 days of index surgery. Substance or medical use disorders evaluated included opioids, long term or current NSAID use, cannabinoids, alcohol, and tobacco use. It was posited that any assertion of substance uses disorders linked to a medical record implies a history of antecedent or simultaneous substance use. Common spine pathologies including degenerative pathologies, spine fractures, spinal cord injury, congenital disorders, inflammatory spondylopathy, osteoporosis and history of prior spine surgery were also evaluated between both the groups.

### Statistical analysis

The incidence of pseudoarthrosis development was determined using a basic bucket creation function built in the Pearldiver database. The average time it took for pseudoarthrosis to develop was calculated using the time-between function in Pearldiver, which determines the average time between the first index procedure claim and the pseudoarthrosis claim. Baseline differences between patients who developed pseudoarthrosis and those who did not within 10 years following lumbar spinal fusion were analyzed using a chi-squared test, and their respective p-values were calculated. All characteristics that were found to be statistically significant were further assessed through multivariate analysis. Each variable significantly different at baseline was controlled for the next variable tested to evaluate if the next variable had a significant association (potential confounding factors). This strategy was conducted for all the variables that were considered predictors of pseudoarthrosis. We further assessed the impact of variables found to be statistically significant on the development of pseudoarthrosis over a 10-year period using Cox proportional hazards survival analysis. For each significant variable, we determined hazard ratios (HR), 95% confidence intervals (CI), and p-values. We also evaluated the proportional hazards assumption for each significant variable using the Schoenfeld Residual Test and reported the associated p-values. Variables with a low p-value (typically <.05) in the Schoenfeld Residual Test were considered to violate the proportional hazards assumption, indicating that the predictor variable's relationship with time is not constant, and these variables were not discussed. The results of the multivariate analyses were reported as odds ratios, while the Cox proportional hazards results were reported as HR with corresponding 95% confidence intervals CI. Hypothesis testing was conducted at a 5% type I error rate (alpha=0.05).

## Results

In this retrospective study, a comprehensive analysis was conducted on a cohort of 76,337 patients who underwent spinal fusion surgery between 2010 and 2022 and were followed for 10 years. Among this patient population, 2.70% (N=2,060) were diagnosed with pseudoarthrosis within the 10 years following their primary fusion surgery. These individuals had an average age of 55.78 years (SD 11.26) and an ECI score of 4.33 (SD 3.77), indicating the average health status at the time of surgery (Table 1). On average, it took 7.38 years (SD 2.03 years) to develop pseudoarthrosis.

**Table 1:** Baseline demographic characteristics of control group patients and those developing pseudoarthrosis within 10 years after lumbar fusion surgery.

	Control N=74,277 (%)	Pseudoarthrosis N=2060 (%)	p-value
Age (years)	56.59	55.78	**<.05**
Gender (M)	30,083 (40.50%)	845 (41.02%)	.65
ECI score	2.45	4.33	**<.05**
Comorbidities			
*Obesity*	31,672 (42.63%)	10,82 (52.52%)	**<.05**
*Vitamin D deficiency*	2004 (2.70%)	128 (6.21%)	**<.05**
*Diabetes*	26,706 (35.98%)	792 (38.44%)	**.02**
*Osteoarthritis*	43,208 (58.20%)	1,417 (68.64%)	**<.05**
Surgical approach			
*Anterior*	14,873 (20.00%)	556 (26.99%)	**<.05**
*Posterior*	32,406 (43.62%)	736 (35.73%)	**<.05**
Surgical levels			
*Single*	39,273 (52.79%)	704 (34.13%)	**<.05**
*2-levels*	11,281 (15.21%)	340 (16.50%)	.11
*3-levels*	14,540 (19.58%)	441 (21.41%)	**.04**
Wound infections	1,595 (2.15%)	69 (3.35%)	**<.05**

Bold values indicate that p-value is less than 0.05.

Upon conducting a chi-square analysis, significant differences emerged when comparing patients who developed symptomatic lumbar pseudoarthrosis within a decade of their lumbar fusion surgery to those who did not. Specifically, these individuals had higher odds of having associated diagnoses of obesity, vitamin D deficiency, diabetes, and osteoarthritis. Furthermore, patients in the pseudoarthrosis group exhibited higher odds of undergoing surgeries that utilized an anterior approach and encompassed 3 or more levels. Additionally, they were more likely to experience wound infections within 30 days postsurgery, implying a potential role of postoperative complications in pseudoarthrosis development (Table 1).

The study also explored the prevalence of drug misuse or abuse, including opioids, long-term NSAID use, tobacco, and alcohol consumption, among the patient population (Table 2). Patients who developed pseudoarthrosis showed significantly higher rates of opioid misuse or abuse (6.74% vs. 2.91%), long-term NSAID use (9.02% vs. 4.61%), and tobacco consumption (56.31% vs. 45.69%) compared to those without pseudoarthrosis. However, cannabinoids and alcohol consumption did not exhibit significant differences between the groups.

**Table 2:** Prevalence of substance use disorder at baseline of control group patients and those developing pseudoarthrosis within 10 years after lumbar fusion surgery.

Substance use disorder	Control N=74,277 (%)	Pseudoarthrosis N=2060 (%)	p-value
Opioid	2,162 (2.91%)	139 (6.74%)	**<.05**
NSAIDs	3,422 (4.61%)	186 (9.02%)	**<.05**
Cannabinoids	566 (0.76%)	19 (0.92%)	.49
Alcohol	5,160 (6.94%)	201 (9.77%)	**<.05**
Tobacco	33,943 (45.69%)	1,162 (56.31%)	**<.05**

Bold values indicate that p-value is less than 0.05.

Furthermore, the presence of various spine-related pathologies was significantly more prevalent among patients who developed pseudoarthrosis (Table 3). These included spine fractures (29.81% vs. 15.00%), congenital disorders (29.42% vs. 17.94%), and inflammatory spondylopathy (19.71% vs. 11.69%). Conversely, diabetes and tobacco use were unexpectedly associated with a lower incidence of symptomatic pseudoarthrosis.

**Table 3:** Prevalence of spinal pathologies at baseline of control group patients and those developing pseudoarthrosis within 10 years after lumbar fusion surgery.

Spinal pathology	Control N=74,277 (%)	Pseudoarthrosis N=2060 (%)	p-value
Degenerative pathologies	71,184 (95.75%)	2,017 (97.92%)	**<.05**
Spine fractures	11,154 (15.00%)	614 (29.81%)	**<.05**
Spinal cord injury	1,210 (1.63%)	58 (2.82%)	**<.05**
Congenital disorders	13,305 (17.94%)	606 (29.42%)	**<.05**
Inflammatory spondylopathy	8,694 (11.69%)	406 (19.71%)	**<.05**
Osteoporosis	9,701 (13.04%)	329 (15.97%)	**<.05**
Surgical aftercare/revision*	46,739 (62.88%)	1,664 (80.78%)	**<.05**

*p-value less than 0.05 is significant.

In a multivariate analysis that controlled for statistically significant baseline variables, it was revealed that factors increasing the risk of pseudoarthrosis included male gender, a higher ECI score, associated diagnoses of vitamin D deficiency, osteoarthritis, an anterior surgical approach, multiple-level surgeries, opioid abuse, and long-term NSAID use (Table 4). Additionally, certain spine disorders, such as spine fractures, congenital disorders, inflammatory spondylopathy, and prior spine surgery, were also linked to an increased risk of pseudoarthrosis. Notably, diabetes and tobacco use were associated with a lower incidence of symptomatic pseudoarthrosis.

**Table 4:** Multivariate regression analysis of predictive factors for the development of pseudoarthrosis in patients undergoing multilevel primary lumbar fusions-controlling for age, gender, and ECI.

	ORs [95% CI]	p-Value
Age (years)	0.99 [0.98, 0.99]	**<.05**
ECI	1.21 [1.19, 1.22]	**<.05**
Obesity	1.05 [0.95, 1.15]	.36
Vitamin D deficiency	1.25 [1.02, 1.52]	**.03**
Diabetes	0.73 [0.66, 0.81]	**<.05**
Osteoarthritis	1.21 [1.09, 1.35]	**<.05**
Anterior	1.17 [1.04, 1.32]	**<.05**
Posterior	0.82 [0.74, 0.92]	**<.05**
Single	0.41 [0.37, 0.46]	**<.05**
2-level	0.59 [0.51, 0.68]	**<.05**
3-level	0.60 [0.53, 0.69]	**<.05**
Wound	0.89 [0.69, 1.15]	.38
Opioids	1.51 [1.25, 1.82]	**<.05**
NSAIDs	1.61 [1.37, 1.89]	**<.05**
Cannabinoids	0.65 [0.41, 1.05]	.08
Alcohol	0.95 [0.81, 1.11]	.51
Tobacco	1.21 [1.10, 1.32]	**<.05**
Degenerative pathologies	1.06 [0.78, 1.45]	.70
Spine fractures	1.57 [1.41, 1.74]	**<.05**
Spinal cord injury	0.97 [0.73, 1.28]	.81
Congenital disorders	1.24 [1.12, 1.38]	**<.05**
Inflammatory spondylopathy	1.17 [1.04, 1.32]	**.01**
Osteoporosis	0.96 [0.84, 1.10]	.55
Surgical aftercare/revision*	1.55 [1.37, 1.74]	**<.05**

*p-value less than 0.05 is significant.

The results of the cox proportional hazards survival analysis of over 10-years (Table 5) showed that vitamin D deficiency (HR=1.29, 95% CI [1.07, 1.56], p=.01) and osteoarthritis (HR=1.21, 95% CI [1.09, 1.35], p<.05) were associated with an increased risk of pseudoarthrosis. Similarly, opioid use (HR=1.44, 95% CI [1.20, 1.73], p<.05), NSAID use (HR=1.51, 95% CI [1.29, 1.77], p<.05), and tobacco use (HR=1.21, 95% CI [1.10, 1.33], p<0.05) were found to also increase the risk of pseudoarthrosis. Notably, only congenital disorders (HR=1.24, 95% CI [1.12, 1.37], p<.05) were linked to an elevated risk of pseudoarthrosis. The proportional hazards assumption was met for all the aforementioned variables.

**Table 5:** Cox proportional hazards analysis of predictive factors for the development of pseudoarthrosis in patients undergoing multilevel primary lumbar fusions.

Risk factor	HR	95% CI [lower limit]	95% CI [upper limit]	p-value	Schoenfeld Residuals test (p-value)
Comorbidities					
Vitamin D deficiency	1.29	1.07	1.56	.01	.91
Diabetes	0.75	0.68	0.83	<.05	.65
Osteoarthritis	1.21	1.09	1.35	<.05	.35
Surgical variables					
Anterior	1.15	1.03	1.30	.02	.02
Posterior	0.84	0.75	0.93	<.05	<.05
Single	0.44	0.39	0.50	<.05	0.05
2-level	0.62	0.54	0.72	<.05	.09
3-level	0.63	0.55	0.72	<.05	.14
Substance use disorders					
Opioids	1.44	1.20	1.73	<.05	.24
NSAIDs	1.51	1.29	1.77	<.05	.41
Tobacco	1.21	1.10	1.33	<.05	.76
Spine pathologies					
Spine fractures	1.59	1.44	1.76	<.05	.001
Congenital disorders	1.24	1.12	1.37	<.05	.24
Inflammatory spondylopathy	1.18	1.05	1.32	<.05	.05
Surgical aftercare/revision	1.58	1.40	1.78	<.05	<.05

## Discussion

The study's findings reveal valuable insights into the incidence and predictive factors of symptomatic lumbar pseudoarthrosis following multilevel lumbar spinal fusion surgery. The observed incidence rate of 2.70% within a 10-year follow-up period, while falling below the commonly reported range of 5% to 35%, is noteworthy. This discrepancy is partly attributed to our specific definition of pseudoarthrosis, which requires a diagnostic claim only when symptoms necessitate professional evaluation or surgery. Consequently, an average time to symptomatic presentation of pseudoarthrosis is 7.38 years. This knowledge is vital for informed patient counseling, as prior education has been shown to enhance patient satisfaction rates with the procedure [16].

Comorbidities such as obesity, vitamin D deficiency, diabetes, and osteoarthritis not only exhibit higher prevalence but also serve as positive predictors of symptomatic pseudoarthrosis. In addition, their impact is consistent thorough out the follow-up period of 10 years. A systematic review of recent literature concerning vitamin D deficiency and spinal fusion outcomes suggests that patients with preoperative vitamin D deficiency achieved lower fusion rates and suffered higher rates of low back pain compared to patients with normal vitamin D levels [17]. Postoperative vitamin D supplementation in deficient patients showed improvements in clinical and functional outcomes, including levels of disability, quality of life, patient-reported surgical outcomes, low back pain, and fusion rates compared to control groups [17]. These findings emphasize the potential benefits of vitamin D supplementation in deficient individuals undergoing primary lumbar fusion surgery. However, the correlation between osteoarthritis and pseudoarthrosis requires further exploration, particularly considering the potential impact of the joint involved with the associated osteoarthritis claim.

The anterior approach for fusion and the involvement of multiple spinal levels have shown a positive linear correlation with the development of symptomatic pseudoarthrosis. Similar observations were made by Madan and Boeree [18], who reported higher rates of successful lumbar fusion with posterior approaches compared to the anterior approach. This observation can be attributed to the fact that fusion surgeries conducted through posterior approaches typically involve a greater number of levels, which leads to fewer independent vertebrae available to accommodate degeneration and subsequently results in an increased risk of pseudoarthrosis development. This is also why surgeries involving multiple levels were also found to be predictive of symptomatic pseudoarthrosis. A study investigating the risk factors for pseudoarthrosis following spinal fusion found that patients with 4 to 8 or ≥9 fused vertebrae had a higher incidence of pseudoarthrosis than those with 2 to 3 fused vertebrae [19].

Substance and medication use disorders, including chronic opioid use, NSAID use, and tobacco use, were found to be positively linked to the development of symptomatic lumbar pseudoarthrosis. These findings align with numerous literature analyses exploring the relationship between opioid use and spinal fusion surgeries, where opioids have been found to downregulate osteoblasts in vitro and have negative effects on bone remodeling and healing by delaying maturation in animal models [20]. Historically, spinal surgeries often resulted in postoperative opioid prescriptions, but recent efforts are focused on finding alternative approaches to pain management in these inherently painful procedures, both to address the opioid epidemic and to reduce the risk of pseudoarthrosis development [21,22].

The literature presents mixed results regarding the correlation between NSAID use and the development of symptomatic pseudoarthrosis [23]. While most animal model studies indicate increased fracturing and delayed healing in cohorts receiving NSAIDs, there is no consensus on the effects of NSAIDs on pseudoarthrosis development in humans [24]. Newer studies suggest that the dose and duration of perioperative NSAID use may influence pseudoarthrosis development, with higher doses and chronic usage correlating with higher rates of nonunion. The study did not establish a correlation between cannabinoid use and pseudoarthrosis development. Literature on preoperative marijuana use in relation to pseudoarthrosis development is limited, with a primary focus on the analgesic effects of marijuana following spine surgery. However, a study comparing clinical and patient-reported outcomes after lumbar fusion surgery in patients who did and did not use marijuana preoperatively found no significant difference in scores, even though marijuana users were typically younger [25]. This suggests that the use of cannabinoids as analgesics in spinal fusion surgeries does not have a long-term impact on pseudoarthrosis development. Moreover, cannabinoids may help reduce the reliance on opioids in these inherently painful procedures. Further research is needed to establish a comprehensive understanding of the correlation between cannabinoid use and pseudoarthrosis development in the context of spinal surgery. This will enable the regulation of the increasing use of marijuana for both medicinal and recreational purposes, ensuring that benefits are maximized while drawbacks are appropriately accounted for. Finally, a history of spinal pathologies requires prior spinal surgery emerged as a predictive factor for pseudoarthrosis development, suggesting that instability [26] resulting from prior surgery may predispose patients to pseudoarthrosis.

Our analysis is subject to several limitations. First, the claims for pseudoarthrosis were collected postprimary surgery. This leaves room for the possibility of other surgical procedures, comorbidities, or traumatic events occurring between the primary surgery and the initial claim for pseudoarthrosis, which could contribute to the development of pseudoarthrosis but were not taken into consideration. While we have established strong correlations between variables due to the substantial number of patients in our study, it's crucial to acknowledge that causation cannot be definitively established. In some instances, a causal relationship can be reasonably inferred based on underlying physiological mechanisms and by cross-referencing our results with those of other studies employing different methodologies. Another noteworthy limitation is the potential presence of undocumented opioid use within our initial population selection, which we were unable to account for. The inherent lack of accountability for such undocumented claims is intrinsic to our study, as is the case with any study relying on insurance claims databases. Additionally, our study is based on the ICD-10 code for pseudoarthrosis, with the ICD-9 code being excluded due to its lack of specificity. This exclusion may lead to an underestimation of pseudoarthrosis prevalence.

In contrast, a significant strength of our study lies in the large number of patients included in the analysis. While retrospective database studies may not provide definitive evidence for establishing associations, our research identifies potential predictors of pseudoarthrosis. This can guide future research endeavors aimed at mitigating healthcare resource allocation disparities. Furthermore, our study sheds light on potential risk factors that, to the best of our knowledge, have not been explored before. These factors include vitamin D deficiency, osteoarthritis, CBD, and a history of other spinal disorders.

## Conclusions

In conclusion, this study found a 2.70% incidence of symptomatic lumbar pseudoarthrosis within 10-year of index procedure with an average 7.38 years leading to its development. Furthermore, this study underscores the potential positive correlation between certain comorbidities, surgical approaches, and substance use disorders, on the development of symptomatic pseudoarthrosis. While the findings align with existing literature, they also introduce novel areas of investigation, such as the impact of vitamin D deficiency and the relationship between cannabinoid use and pseudoarthrosis. Further research in these areas can lead to more effective patient counseling, improved surgical planning, and enhanced outcomes for patients undergoing lumbar spinal fusion surgery.

## Declarations of competing interests

The authors declare that they have no known competing financial interests or personal relationships that could have appeared to influence the work reported in this paper.

## Appendix Supplementary materials

Image, application 1
