# Supplementary material for: Predictive factors of symptomatic lumbar pseudoarthrosis following multilevel primary lumbar fusion
Source: N Am Spine Soc J. 2023 Dec 5;17:100302. doi: 10.1016/j.xnsj.2023.100302 (PMC10844967; doi:10.1016/j.xnsj.2023.100302)
Supplement: Supplementary file 1 [file mmc1.docx]

**Appendix I**

|  |  |
| --- | --- |
| Lumbar fusions | CPT-22612, CPT-22630, CPT-22633, CPT-22558 |
| Pseudoarthrosis | ICD-10-D-M960 |
| Vitamin D deficiency | ICD-9-D-2689, ICD-10-D-E559 |
| Wound complications | ICD-9-D-99811, ICD-9-D-99813, ICD-9-D-99812, ICD-9-D-99830, ICD-9-D-99831, ICD-9-D-99832, ICD-9-D-99833, ICD-9-D-99883, ICD-9-D-9985, ICD-9-D-9986, ICD-9-D-9987, ICD-9-D-9988 |
| Long-term or current NSAID use | ICD-10-D-Z791 |
|  |  |
| Spine Disorders | |
| Degenerative spine diseases | ICD-9-D-8460, ICD-9-D-8461, ICD-9-D-8462, ICD-9-D-8463, ICD-9-D-8468, ICD-9-D-8469, ICD-9-D-847, ICD-9-D-8479, ICD-9-D-8472, ICD-9-D-8470, ICD-9-D-723, ICD-9-D-7238, ICD-9-D-7231, ICD-9-D-7210, ICD-9-D-7211, ICD-9-D-7212, ICD-9-D-7213, ICD-9-D-7214, ICD-9-D-7217, ICD-9-D-7218, ICD-9-D-7219, ICD-9-D-72190, ICD-9-D-72191, ICD-9-D-7224, ICD-9-D-7225, ICD-9-D-72251, ICD-9-D-72252, ICD-9-D-7226, ICD-9-D-72290, ICD-9-D-72292, ICD-9-D-72291, ICD-9-D-72293, ICD-9-D-7242, ICD-9-D-7245, ICD-9-D-7246, ICD-9-D-72470, ICD-9-D-72471, ICD-9-D-72479, ICD-9-D-7248, ICD-9-D-7249, ICD-9-D-7220, ICD-9-D-72210, ICD-9-D-72211, ICD-9-D-7222, ICD-9-D-3539, ICD-9-D-3550, ICD-9-D-3559, ICD-9-D-72270, ICD-9-D-72271, ICD-9-D-72272, ICD-9-D-72273, ICD-9-D-7243, ICD-9-D-7214, ICD-9-D-7244, ICD-9-D-72142, ICD-9-D-72191, ICD-9-D-72400, ICD-9-D-72402, ICD-9-D-72409, ICD-9-D-7230, ICD-9-D-72401, ICD-9-D-7384, ICD-9-D-75611, ICD-9-D-75612, ICD-9-D-7384, ICD-9-D-75611, ICD-9-D-75612, ICD-9-D-73739, ICD-9-D-7373, ICD-9-D-73730, ICD-9-D-737, ICD-9-D-73720, ICD-9-D-7378, ICD-9-D-7379, ICD-9-D-7371, ICD-9-D-73710, ICD-9-D-73719, ICD-9-D-73732, ICD-9-D-73734, ICD-9-D-73743, ICD-9-D-3539, ICD-9-D-3550, ICD-9-D-3559, ICD-9-D-7384, ICD-9-D-75611, ICD-9-D-75612, ICD-9-D-73739, ICD-9-D-7373, ICD-9-D-73730, ICD-9-D-737, ICD-9-D-73720, ICD-9-D-7378, ICD-9-D-7379, ICD-9-D-7371, ICD-9-D-73710, ICD-9-D-73719, ICD-9-D-73732, ICD-9-D-73734, ICD-9-D-73743, ICD-9-D-7331, ICD-9-D-73310, ICD-9-D-73313, ICD-9-D-7338, ICD-9-D-73381, ICD-9-D-73382, ICD-9-D-73395, ICD-9-D-805, ICD-9-D-8050, ICD-9-D-80500, ICD-9-D-80501, ICD-9-D-80502, ICD-9-D-80503, ICD-9-D-80504, ICD-9-D-80505, ICD-9-D-80506, ICD-9-D-80507, ICD-9-D-80508, ICD-9-D-8051, ICD-9-D-80510, ICD-9-D-80511, ICD-9-D-80512, ICD-9-D-80513, ICD-9-D-80514, ICD-9-D-80515, ICD-9-D-80516, ICD-9-D-80517, ICD-9-D-80518, ICD-9-D-8052, ICD-9-D-8053, ICD-9-D-8054, ICD-9-D-8055, ICD-9-D-8056, ICD-9-D-8057, ICD-9-D-8058, ICD-9-D-8059, ICD-9-D-806, ICD-9-D-8060, ICD-9-D-80600, ICD-9-D-80601, ICD-9-D-80602, ICD-9-D-80603, ICD-9-D-80604, ICD-9-D-80605, ICD-9-D-80606, ICD-9-D-80607, ICD-9-D-80608, ICD-9-D-80609, ICD-9-D-8061, ICD-9-D-80610, ICD-9-D-80611, ICD-9-D-80612, ICD-9-D-80613, ICD-9-D-80614, ICD-9-D-80615, ICD-9-D-80616, ICD-9-D-80617, ICD-9-D-80618, ICD-9-D-80619, ICD-9-D-8062, ICD-9-D-80620, ICD-9-D-80621, ICD-9-D-80622, ICD-9-D-80623, ICD-9-D-80624, ICD-9-D-80625, ICD-9-D-80626, ICD-9-D-80627, ICD-9-D-80628, ICD-9-D-80629, ICD-9-D-8063, ICD-9-D-80630, ICD-9-D-80631, ICD-9-D-80632, ICD-9-D-80633, ICD-9-D-80634, ICD-9-D-80635, ICD-9-D-80636, ICD-9-D-80637, ICD-9-D-80638, ICD-9-D-80639, ICD-9-D-8064, ICD-9-D-8065, ICD-9-D-8066, ICD-9-D-80660, ICD-9-D-80661, ICD-9-D-80662, ICD-9-D-80669, ICD-9-D-8067, ICD-9-D-80670, ICD-9-D-80671, ICD-9-D-80672, ICD-9-D-80679, ICD-9-D-8068, ICD-9-D-8069, ICD-9-D-8390, ICD-9-D-83900, ICD-9-D-83901, ICD-9-D-83902, ICD-9-D-83903, ICD-9-D-83904, ICD-9-D-83905, ICD-9-D-83906, ICD-9-D-83907, ICD-9-D-83908, ICD-9-D-8391, ICD-9-D-83910, ICD-9-D-83911, ICD-9-D-83912, ICD-9-D-83913, ICD-9-D-83914, ICD-9-D-83915, ICD-9-D-83916, ICD-9-D-83917, ICD-9-D-83918, ICD-9-D-8392, ICD-9-D-83920, ICD-9-D-83921, ICD-9-D-8393, ICD-9-D-83930, ICD-9-D-9051, ICD-9-D-V5417, ICD-9-D-V5427 |
| Spinal Fractures | ICD-9-D-7331, ICD-9-D-73310, ICD-9-D-73313, ICD-9-D-7338, ICD-9-D-73381, ICD-9-D-73382, ICD-9-D-73395, ICD-9-D-805, ICD-9-D-8050, ICD-9-D-80500, ICD-9-D-80501, ICD-9-D-80502, ICD-9-D-80503, ICD-9-D-80504, ICD-9-D-80505, ICD-9-D-80506, ICD-9-D-80507, ICD-9-D-80508, ICD-9-D-8051, ICD-9-D-80510, ICD-9-D-80511, ICD-9-D-80512, ICD-9-D-80513, ICD-9-D-80514, ICD-9-D-80515, ICD-9-D-80516, ICD-9-D-80517, ICD-9-D-80518, ICD-9-D-8052, ICD-9-D-8053, ICD-9-D-8054, ICD-9-D-8055, ICD-9-D-8056, ICD-9-D-8057, ICD-9-D-8058, ICD-9-D-8059, ICD-9-D-806, ICD-9-D-8060, ICD-9-D-80600, ICD-9-D-80601, ICD-9-D-80602, ICD-9-D-80603, ICD-9-D-80604, ICD-9-D-80605, ICD-9-D-80606, ICD-9-D-80607, ICD-9-D-80608, ICD-9-D-80609, ICD-9-D-8061 ICD-9-D-80610, ICD-9-D-80611, ICD-9-D-80612, ICD-9-D-80613, ICD-9-D-80614, ICD-9-D-80615, ICD-9-D-80616, ICD-9-D-80617, ICD-9-D-80618, ICD-9-D-80619, ICD-9-D-8062, ICD-9-D-80620, ICD-9-D-80621, ICD-9-D-80622, ICD-9-D-80623, ICD-9-D-80624, ICD-9-D-80625, ICD-9-D-80626, ICD-9-D-80627, ICD-9-D-80628, ICD-9-D-80629, ICD-9-D-8063, ICD-9-D-80630, ICD-9-D-80631, ICD-9-D-80632, ICD-9-D-80633, ICD-9-D-80634, ICD-9-D-80635, ICD-9-D-80636, ICD-9-D-80637, ICD-9-D-80638, ICD-9-D-80639, ICD-9-D-8064, ICD-9-D-8065, ICD-9-D-8066, ICD-9-D-80660, ICD-9-D-80661, ICD-9-D-80662, ICD-9-D-80669, ICD-9-D-8067, ICD-9-D-80670, ICD-9-D-80671, ICD-9-D-80672, ICD-9-D-80679, ICD-9-D-8068, ICD-9-D-8069, ICD-9-D-8390, ICD-9-D-83900, ICD-9-D-83901, ICD-9-D-83902, ICD-9-D-83903, ICD-9-D-83904, ICD-9-D-83905, ICD-9-D-83906, ICD-9-D-83907, ICD-9-D-83908, ICD-9-D-8391, ICD-9-D-83910, ICD-9-D-83911, ICD-9-D-83912, ICD-9-D-83913, ICD-9-D-83914, ICD-9-D-83915, ICD-9-D-83916, ICD-9-D-83917, ICD-9-D-83918, ICD-9-D-8392, ICD-9-D-83920, ICD-9-D-83921, ICD-9-D-8393, ICD-9-D-83930, ICD-9-D-9051, ICD-9-D-V5417, ICD-9-D-V5427 |
| Spinal cord injury | ICD-9-D-3369, ICD-9-D-9520, ICD-9-D-95200, ICD-9-D-95203, ICD-9-D-95205, ICD-9-D-95209, ICD-9-D-95210, ICD-9-D-9529, ICD-9-D-9530, ICD-9-D-95204 |
| Congenital spine disorders | ICD-9-D-3241, ICD-9-D-34460, ICD-9-D-7215, ICD-9-D-7216, ICD-9-D-7217, ICD-9-D-72230, ICD-9-D-72232, ICD-9-D-7237, ICD-9-D-7246, ICD-9-D-72740, ICD-9-D-73320, ICD-9-D-7385, ICD-9-D-7393, ICD-9-D-7394, ICD-9-D-74190, ICD-9-D-7542, ICD-9-D-75610, ICD-9-D-75613, ICD-9-D-75614, ICD-9-D-75615, ICD-9-D-75616, ICD-9-D-75617, ICD-9-D-75619 |
| Inflammatory spondylopathy | ICD-9-D-7200, ICD-9-D-7201, ICD-9-D-7202, ICD-9-D-7208, ICD-9-D-72081, ICD-9-D-72089, ICD-9-D-7209 |
| Osteoporosis | ICD-9-D-7330, ICD-9-D-73300, ICD-9-D-73301, ICD-9-D-73302, ICD-9-D-73303, ICD-9-D-73309, ICD-9-D-V1781, ICD-9-D-V8281 |
| Prior spine surgery | ICD-9-D-72280, ICD-9-D-72281, ICD-9-D-72283, ICD-9-D-8749, ICD-9-D-99667, ICD-9-D-E8781, ICD-9-D-9962, ICD-9-D-99659, ICD-9-D-9964, ICD-9-D-99640, ICD-9-D-99641, ICD-9-D-99649, ICD-9-D-99859, ICD-9-D-V454, ICD-9-D-99678 |
